# Spontaneous rupture of the spleen operated in gynecological unit mistaken for ruptured hemorrhagic ovarian cyst: total splenectomy

**DOI:** 10.11604/pamj.2014.19.324.5749

**Published:** 2014-11-26

**Authors:** Filbert Eko Eko, Florent Ymele Fouelifack, Elanga Vincent de Paul

**Affiliations:** 1 Obstetrics and Gynecology Unit, Yaoundé Central Hospital, Cameroon; 2 Visceral Surgeon, Yaounde Central Hospital, Cameroon

**Keywords:** Spleen, rupture, splenectomy

## Abstract

Spontaneous splenic rupture is always neglected when consulting acute abdominal pains in gynecological emergencies. It constitutes about 1% of all splenic ruptures and can be managed by abstention, surgery or embolization. We present the case of a young lady who was diagnosed of spontaneous rupture during surgery that was mistaken for ruptured hemorrhagic ovarian cyst and finally treated by total splenectomy. The pre-operative work up was absolute for a rupturred hemorrhagic cyst and secondariy for a ruptured ectopic gestation.

## Introduction

Abdominal pain is one of the most common reasons for emergency department visits. Splenic rupture, which is a life-threatening abdominal emergency, constitutes a group among causes of abdominal pain, and is often seen as secondary to abdominal trauma. It requires rapid diagnosis and treatment. Spontaneous rupture of the spleen is a rare condition and constitutes up to 1% of all splenic ruptures [1]. It can be mistakenly managed since there are many differential diagnoses. We hereby present a case of fortuitous discovery of splenic rupture during laparotomy performed for ruptured hemorrhagic ovarian cyst, in a Cameroonian woman.

## Patient and observation

Mrs XY. is 23 year old, nulliparous woman who was wheeled to the emergency service of the gynaeco-obstetrical unit of the hospital for severe abdominal pains of sudden unset followed by dizziness and loss of stability leading to a fall and collapsed. She has no amenorrhea and her hemoglobin electrophoresis is AA. She has no particular medical or surgical past history. Interrogation reveals her last coitus some minutes before onset of symptoms after 2years of forced abstinence (fiancé abroad), no accident or fall, and no fight. Physical examination reveals conserved general state, unstable hemodynamic parameters (BP 70/50mmHg, pulse 100bpm, temperature 37.3°C). Her conjunctivae were coloured with a distended abdomen especially in the pelvic region of hemoperitoneum confirmed by an abdominal tap (7milliliters of unclotted blood). Vaginal exams (digital and speculum) reveal no bleeding, no pelvic mass but tender with bulging pouch of Douglas.

**Figure 1:**
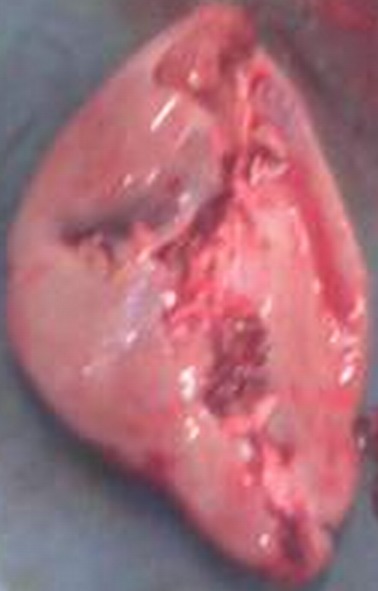
The ruptured spleen after total splenectomy

A presumptive working diagnosis of a ruptured hemorrhagic ovarian cyst was considered. An emergency pre-operative work-up (FBC, PT, and CKT) was performed as routine and she was prepared for exploratory laparotomy under general anesthesia. Two units (500 milliliters /unit) of Blood were also reserved. During surgery, we found hemoperitoneum of about 2500 milliliters of fresh blood, a clean pelvis with neither adhesions nor a pathologic tube or ovary. Extension of the incision anteriorly for abdominal exploration reveals a macroscopically healthy liver but the spleen presents lacerations at 3 points posteriorly (2-4cm) that were actively bleeding (grade 3). Total splenectomy was done (Figure 1) with the help of abdominal surgeons. Peritoneal toileting with closure of the abdominal wall followed. Her post-operative period was uneventful on antibiotics and antalgics until her discharge after 7days of hospitalization. Neither pathological examination of the splenectomy material nor postoperative consultations with the hematology and infectious diseases departments showed any pathology to explain the rupture. Vaccination against certain predisposed infections (like pneumococcus infection was also advised.

## Discussion

The first published case of spontaneous rupture of the spleen was by Rokitansky in 1861 [2]. It was later described in 1874 by the english surgeon Atkinson [3]. His publication was followed by numerous case reports until a review of all these cases was published in 1958, which was the most comprehensive study at that time [4]. They concluded that “a small number of cases which, when subjected to careful scrutiny, defy any conclusion other than that they represent instances of spontaneous rupture of the normal spleen”. For spontaneous rupture of the spleen, there is often an underlying pathology such as an infectious disease (especially Epstein-Barr virus, hepatitis, salmonella, malaria), a neoplastic disease (lymphoma, leukemia), or a hematological or connective tissue disease [5].

Typically, patients present with hypovolemic shock and signs of peritonitis. Two other signs that are suggestive of splenic rupture are Kehr's sign (left diaphragmatic irritation resulting in referred pain to the left shoulder) and Balance's sign (palpable tender mass in the left upper quadrant) [6]. None of these were present in our patient. Her pain was at the left iliac region irradiating to the back. It may be accompanied with nausea, vomiting, dizziness, and syncope. Patients may have signs of shock like in our case but the difficulty in diagnosis was obvious because of the localization of the pain. Radiological imaging can play an important role in diagnosing when a ruptured spleen is suspected. Plain abdominal radiographs can show enlargement of the spleen with medial displacement of the gastric bubble and elevation of the left hemidiaphragm with impaired motion [7]. Angiographic findings include extravasation of contrast, a “mottled” parenchymal phase and arteriovenous shunts with early venous filling and bowing, stretching and crowding of the vessels [7]. Computed tomography Scan (CT scan) is important for staging the splenic rupture and in deciding surgery. It has a sensitivity and specificity of at least 95% in detecting splenic injury [8].

Our case had no trauma history. CT scan was not done because we did not think about rupture of the spleen before surgery. Pre and postoperative hematological and infectious causes were surveyed, and no positive findings were detected except for some viral markers that weren't looked for. Microscopic and macroscopic examinations of the spleen were normal. Therefore, the case was accepted as spontaneous rupture of the spleen. We however had a worry whether it could be post coital according to the history of onset of the pain although she didn't accept a traumatic intercourse. Many theories have been proposed to explain spontaneous rupture of the spleen. Existence of a mall area of localized disease with no traces left on the spleen is an accepted theory. Other theories are quietly chronic passive congestion causes, degenerative changes of the splenic artery or rapid dissection into the splenic parenchyma that triggers the rupture. Cases of spontaneous rupture of the spleen have been described after vomiting [5] and after coughing [6]. In our case, a post coital history is noted which the victim doesn't evaluate as traumatic.

Historically the treatment of choice for all kinds of splenic rupture used to be splenectomy. Nowadays it remains subject to debate. Splenectomy remains the treatment of choice in patients with a hemoperitoneum and severe hypovolemic shock. During laparotomy repair of the spleen or tamponade by the use of an absorbable mesh (splenorraphy) should be considered to preserve splenic tissue [9]. The third possibility (after emergency transcatheter arterial embolization and surgery) when treating splenic rupture is a conservative approach. This is a good option when patients are hemodynamically stable. Conservative management consists of observation for 7 to 14 days in the hospital, strict bed rest and administration of fluid and blood as needed. Rupture usually occurs within the first week of illness, although it may happen in any time of the course of the disease (1-30 days). Stephenson and DuBois conclude in a recent report that patients are considered suitable for the conservative approach when they require less than 4 units or 40 ml/kg of blood for resuscitation [10]. It remains unclear how to follow these patients and whether the pathologic spleen can heal and retain its function. The treatment of hemodynamic stable patients with spontaneous splenic ruptures should be assessed individually with a low threshold for surgery or arterial embolization.

## Conclusion

With this case, we emphasize that spontaneous rupture of the spleen must be considered in the evaluation of abdominal pain in both traumatic and non-traumatic patients. A negative pregnancy test in the context of severe abrupt abdominal pain and hemoperitoneum should initiate thoughts of a ruptured spleen since early recognition and treatment can prevent serious morbidity and mortality.

## References

[1] Badenoch DF, Maurice HD, Gilmore OJA (1985). Spontaneous rupture of a normal spleen. JR Coll Surg Edinb..

[2] Lieberman ME, Levitt A (1989). Spontaneous rupture of the spleen. Am J Emerg Med..

[3] Atkinson E (1874). Death from an idiopathic rupture of the spleen. BMJ..

[4] Orloff MJ, Peskin GW (1958). Spontaneous rupture of the normal spleen: a surgical enigma. Int Abstr Surg..

[5] Lemon M, Dorsch M, Street K, Cohen R, Hale P (2001). Splenic rupture after vomiting. J R Soc Med..

[6] Wehbe E, Raffi S, Osborne D (2008). Spontaneous splenic rupture precipitated by cough: A case report and a review of the literature. Scand J Gastroenterol..

[7] Love L, Greenfield GB, Braun TW, Moncada R, Freeark RJ, Baker RJ (1968). Angiography of splenic trauma. Radiology..

[8] Jeffrey RB, Laing FC, Federle MP, Goodman PC (1981). Computed tomography of splenic trauma. Radiology..

[9] Leemans R, van Mourik JB (1988). A spleen-preserving method in splenic rupture using an absorbable net. Ned TijdschrGeneeskd..

[10] Stephenson JT, DuBois JJ (2007). Nonoperative management of spontaneous splenic rupture in infectious mononucleosis: a case report and review of the literature. Pediatrics..

